# NMDA Receptors of Gastric-Projecting Neurons in the Dorsal Motor Nucleus of the Vagus Mediate the Regulation of Gastric Emptying by EA at Weishu (BL21)

**DOI:** 10.1155/2012/583479

**Published:** 2012-05-13

**Authors:** Xin Zhang, Bin Cheng, Xianghong Jing, Yongfa Qiao, Xinyan Gao, Huijuan Yu, Bing Zhu, Haifa Qiao

**Affiliations:** ^1^Neuroscience Program, Shandong University of Traditional Chinese Medicine, Changqing University Park, Jinan 250355, China; ^2^Institute of Acupuncture and Moxibustion, China Academy of Chinese Medical Sciences, 16 Nanxiaojie, Dongzhimeinei, Beijing 100700, China; ^3^Qingdao Haici Medical Group, 4 Renmin Road, Qingdao 266033, China; ^4^Department of Biomedical Sciences, The Florida State University College of Medicine, 1115 West Call Street, Tallahassee, FL32306, USA

## Abstract

A large number of studies have been conducted to explore the efficacy of electroacupuncture (EA) for the treatment of gastrointestinal motility. While several lines of evidence addressed the basic mechanism of EA on gastrointestinal motility regarding effects of limb and abdomen points, the mechanism for effects of the back points on gastric motility still remains unclear. Here we report that the NMDA receptor (NMDAR) antagonist kynurenic acid inhibited the gastric emptying increase induced by high-intensity EA at BL21 and agonist NMDA enhanced the effect of the same treatment. EA at BL21 enhanced NMDAR, but not AMPA receptor (AMPAR) component of miniature excitatory postsynaptic current (mEPSC) in gastric-projecting neurons of the dorsal motor nucleus of the vagus (DMV). In sum, our data demonstrate an important role of NMDAR-mediated synaptic transmission of gastric-projecting DMV neurons in mediating EA at BL21-induced enhancement of gastric emptying.

## 1. Introduction

Functional gastrointestinal disorders are frequently encountered in ordinary clinical practice. A large number of studies have been conducted to explore the efficacy of electroacupuncture (EA) for the treatment of gastrointestinal motility disorders [1–8]. However, the mechanism of acupuncture on gastrointestinal (GI) motility remains to be clarified. Weishu (BL21), as one of the most common points for gastrointestinal disorders, can accelerate gastric motility [9]. Previous reports demonstrated that EA at various frequencies (1–100 Hz) may stimulate the somatic afferent nerves innervating the skin and muscles of the body and different stimulation procedures are able to excite different somatic afferent fibers and regulate gastric motility through different mechanisms [5, 10, 11].

The inhibitory cutaneojejunal reflex is apparently propriospinal, since it existed in spinal animals, whereas the facilitatory reflex could be supraspinal [11]. The dorsal motor nucleus of the vagus (DMV) plays a critical role in regulation of gastric motility. Receptors of glutamate and GABA in the dorsal vagal complex are involved in regulation of GI motility. GABAA and NMDA receptors are colocalized within DMV neurons projecting to the stomach [12]. Microinjection of glutamate into the DMV enhanced gastric motility [13–15]. On the other hand, microinjection of GABAA antagonists, bicuculline, into the DMV induces dose-dependent increases in gastric motility and secretion [16–18]. Microinjection of GABAA agonist, muscimol, into the DMV abolished bicuculline-induced increases in gastric motility and gastric acid secretion [17, 18]. GABAB receptor antagonist, baclofen, induced outward current of the gastric-projecting neurons at the DMV [19]. EA stimulates glutaminergic neurons in the brainstem resulting in improvement of stress-induced delay of gastric emptying [20]. In current works, we investigated the functional role of NMDA receptors (NMDARs) and GABA receptors (GABARs) in this process and found that high-intensity EA at BL21 enhanced gastric emptying through NMDARs of DMV.

## 2. Materials and Methods

### 2.1. Animals

Male Sprague Dawley (180–200 g) rats were purchased from the Institute of Laboratory Animal Sciences, CAMS & PUMC (Beijing, China), and the Shandong Laboratory animals Center. In this study, all manipulations and procedures were carried out in accordance with The Guide for Care and Use of Laboratory Animals issued by USA National Institutes of Health and were approved by the Institutional Animal Care and Use Committee of China Academy of Chinese Medical Sciences, as well as the Animal Care and Use Committee of Shandong University of Traditional Chinese Medicine. Rats were housed (23 ± 1°C) in groups and maintained under a 12 hours light/dark cycle with food and water available *ad libitum *[21, 22].

### 2.2. Gastric Emptying Study

As described previously [23], After 24 h of fasting, rats were given preweighed pellets (1.5 g) for 10 min. After the start of feeding (10 min), the residual pellets were withdrawn. Food intake was measured by weighing the spilled and uneaten pellets. Rats were killed by decapitation 90 min after the start of feeding under the ketamine (80 mg/kg) and xylazine (8 mg/kg) anesthesia. The stomach was surgically isolated and removed. The gastric content was recovered from the stomach, dried, and weighed. Solid gastric emptying was calculated according to the following formula

Gastric emptying (%) = (1 − (dried weight of food recovered from stomach)/weight of food intake) × 100 [23].

### 2.3. Microinjection in DMV

A glass micropipette (ID 0.04 mm, OD 0.12 mm, WPI, USA) with a tip diameter of ~30 *μ*m was stereotaxically implanted at 0.1 to 0.6 mm rostral to calamus scriptorius (CS), 0.3 to 0.6 mm lateral from the midline, and 0.5 to 0.9 mm below the dorsal surface of the medulla. Microinjections of glutamate or GABA receptor (GABAR) antagonist were performed bilaterally via a Hamilton syringe connected to the micropipette, with the movement of the meniscus monitored by a dissecting microscope. Injections were given in volumes of 5 *μ*L over a period of 45–60 seconds. EA-evoked responses were repeated 10 min after the dorsal motor nucleus of the vagus (DMV) microinjection. The locations of microinjection were confirmed by histological verification.

### 2.4. Histological Verification of Injection Sites

The microinjection site in the brainstem was marked by pontamine sky blue. After fixing in vivo with 2% paraformaldehyde and 1% glutaraldehyde in 0.1 M PBS (pH 7.4), the brainstem was sectioned at 30 *μ*m, and the sections were stained with 0.3% neutral red. The marked microinjection site was located by microscopic examination. Only those data with histological and chemical confirmation were accepted.

### 2.5. Electroacupuncture Stimulation

BL21 was determined by anatomical marks based on the description in textbooks and previous reports [24]. Briefly, BL21 is located on the back, below the spinous process of the 12th thoracic vertebra, 1.5 cun lateral to the posterior midline (about 8 mm in rats). 10 min after drug administration, BL21 was bilaterally stimulated with a 2-3 mA pulse of 1 ms duration at a frequency of 50 Hz for 15 minutes. The stimulation intensity of a strong nociceptive stimulation which can activate C-fiber (7.68 ± 0.53 mA) was given by a pair of needle electrodes inserted 3 mm depth into the skin. The control acupoint Zhongwan (CV12) located on the median line of the upper abdomen, 15 mm above the umbilicus [22], was also inserted to a depth of 3 mm and stimulated with the same above mentioned intensity. The electrical current for EA was generated by a stimulator (Master-8, Israel).

### 2.6. Retrograde Labeling

The crystals of retrograde neuronal tracer 1,1′-dioctadecyl-3,3,3′,3′-tetramethylindocarbocyanine perchlorate (DiIC_18_(3); DiI) (Molecular Probes, Eugene, USA) was used to label gastric-projecting neurons of the DMV in 14-day-old male Sprague Dawley rats (Institute of Laboratory Animal Sciences, CAMS & PUMC, Beijing, China). As described previously [25, 26], after anesthetizing deeply with ketamine/xylazine and performing an abdominal laparotomy, DiI crystals were applied to one gastric region per rat (either the major curvature of the fundus or corpus or the antrum-pylorus). To confine the site of application, the crystals were embedded to the application site using a fast-settling epoxy resin that was allowed to harden for several minutes. After closing the laparotomy with 5/0 suture, the animal was placed in the chamber warmed under a radiant heat lamp until normal activity was restored. The animals were then returned to their home cages and allowed to recover for 10–15 days before brain slices were collected.

### 2.7. Brain Slice Preparation

Thin brainstem slices were prepared from retrograde-labeled rats as described previously with several modifications [25, 26]. Briefly, the rat was sacrificed after being deeply anesthetized with ketamine/xylazine. The whole brain was then removed and placed in ice-cold artificial cerebrospinal fluid (ACSF) containing (in mM): 124 NaCl, 3 KCl, 1.25 NaH_2_PO_4_, 1.3 MgSO_4_, 2 CaCl_2_, 26 NaHCO_3_, 10 glucose, bubbled with 95% O_2_/5% CO_2_, osmolality 300–310 mOsm. After removing the cerebellum, the brainstem was transected rostrally at the level of the pons and again at a point several millimeters caudal to the CS. A vibratome (VT1200S, Leica, German) was used to cut four to five coronal slices (250 *μ*m thickness) containing the DMV. The slices were incubated at 37°C for at least 45 minutes in oxygenated ACSF before use.

### 2.8. Whole-Cell Recording

A single slice was transferred to the recording chamber and kept in place with a slice anchor (Warner Instruments, Hamden, USA). The retrograde-labeled DMV neurons were identified under a Nikon E600 microscope (Nikon, Tokyo, Japan) equipped with tetramethylrhodamine isothiocyanate epifluorescence filters. Electrophysiological recordings were made under brightfield illumination after the identity of a labeled neuron was confirmed. The slice was continuously superfused with oxygenated ACSF (2 mL/min) at room temperature. Recording solution containing (in mM): 145 K-gluconate, 7.5 KCl, 9 NaCl, 1 MgSO_4_, 10 HEPES, 0.2 EGTA, 2 Na-ATP, 0.25 Na-GTP, adjusted to pH 7.4 with KOH, osmolality 290–300 mOsm, was used to back-fill recording electrodes (DC resistance: 5–7 MΩ). Miniature excitatory postsynaptic currents (mEPSCs) were recorded with an Axopatch 200B amplifier (Molecular Devices, USA) and filtered at 2 kHz with a lowpass filter, and data were digitized at 10 kHz and stored online using the pClamp 9 software. The perfusion solution contained 30 *μ*M bicuculline and 1 *μ*M TTX, and the membrane was held at −60 mV. Each data is averaged with 100 events at least. Data were analyzed with the Mini Analysis program (Synaptosoft, Leonia, USA) and WinWCP software (Strathclyde Institute of Pharmacy and Biomedical Sciences, Canada).

### 2.9. Data Analysis

Data was shown as mean ± SEM. For significance evaluation, data sets with normal distribution were analyzed by unpaired *t*-test for two groups or one-way ANOVA followed by *q* test or Dunnett's test for more than two groups, and *P* < 0.05 was considered statistically significant.

## 3. Results

### 3.1. The Effect of EA at BL21 on Gastric Emptying

**Figure 1:**
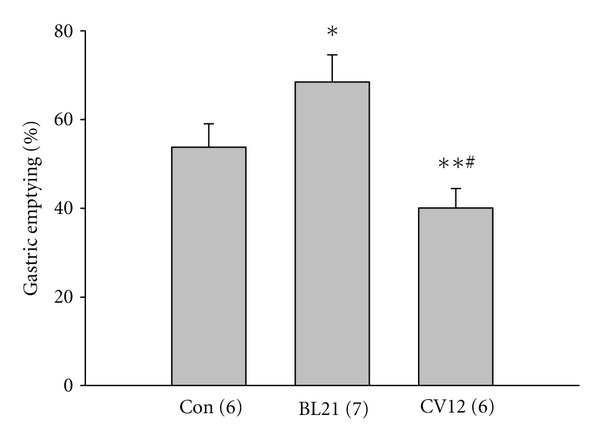
Effects of electroacupuncture (EA) at BL21 and CV12 on gastric emptying. EA at BL21 increases gastric emptying significantly compared to the control (*P < 0.05, **P < 0.01, one-way ANOVA followed by *q* test, ^#^ *P* < 0.05, compared to BL21).

To determine whether EA at BL21 may affect gastric emptying in rats, we designed an experiment in which EA with a stimulation protocol mentioned above was applied at BL21 and selected an abdomen point CV12 as a control it which same stimulation protocol was performed. As shown in Figure 1, EA at BL21 increased gastric emptying capacity (control: 53.80 ± 5.23%; BL21: 68.45 ± 6.12%, *P* < 0.05). vOppositely, EA at CV12 reduced gastric emptying (40.08 ± 4.37%, *P* < 0.05). 

### 3.2. The Role of GABA Receptors in Regulation of Gastric Motility by EA at BL21 Point

**Figure 2:**
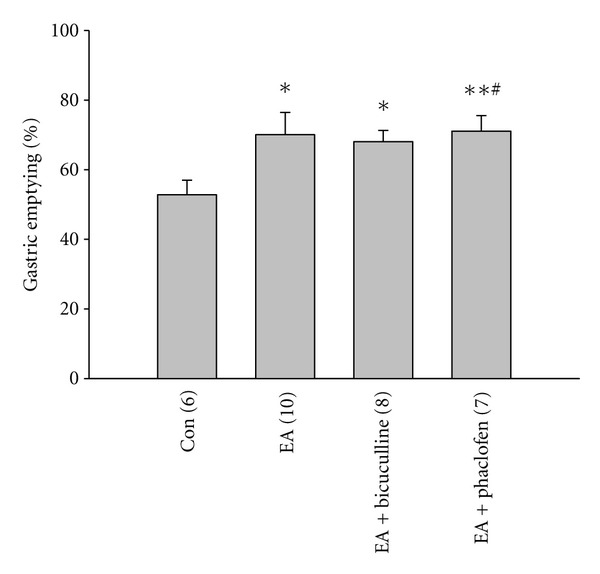
Effects of GABA receptor (GABAR) antagonists, bicuculline and phaclofen, on the regulation of gastric emptying by EA at BL21. EA at BL21 increased gastric emptying significantly but GABAA and GABAB antagonists, bicuculline and phaclofen, did not significantly affect the upregulation of gastric emptying induced by EA at BL21 (*P < 0.05, **P < 0.01, compared to control, ^#^ *P* < 0.05, compared to EA, one-way ANOVA followed by *q* test).

To identify the role of GABA receptors in regulation of gastric emptying by electrostimulation at BL21, we stereotaxically microinjected GABA receptor antagonists bicuculline (for GABAA, 5 *μ*L, 12 nM) and phaclofen (for GABAB, 5 *μ*L, 100 nM) through a glass micropipette into DMV 10 min before EA application. Same volume normal saline was microinjected into DMV in control group. After EA for 15 min, the animals were deeply anesthetized with ketamine/xylazine anesthesia, and the stomach was then isolated and removed. The gastric content was dried and weighted. As shown in Figure 2, compared to the control (52.80 ± 4.18%), EA increased gastric emptying significantly (70.08 ± 6.37%, *P* < 0.05). However, the emptying capacity enhanced by EA did not change significantly after injection of bicuculline or phaclofen (EA ± bicuculline: 68.07 ± 3.24%; phaclofen: 71.09 ± 4.45%, *P* > 0.05), suggesting that the emptying capacity increased by EA at BL21 might not be mediated by GABA receptors of DMV.

### 3.3. The Role of NMDA Receptors in Regulation of Gastric Motility by EA at BL21 Point

**Figure 3:**
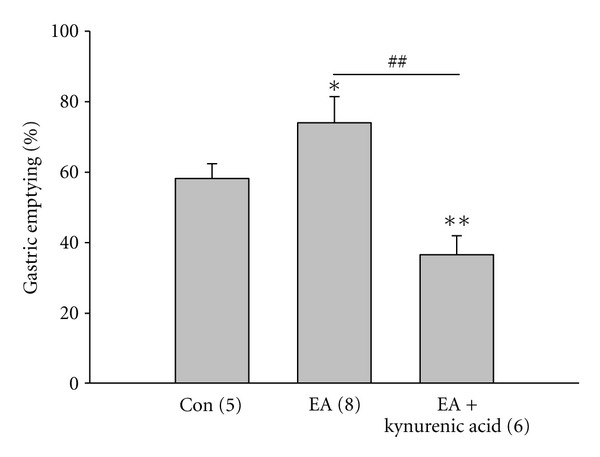
Effect of NMDA receptor (NMDAR) antagonist kynurenic acid on the regulation of gastric emptying by EA at BL21 EA at BL21 increased gastric emptying significantly but NMDAR antagonist kynurenic acid abolished the enhancement of gastric emptying caused by EA at BL21 (*P < 0.05, **P < 0.01, compared to the control; ^##^ *P* < 0.01, compared to EA; one-way ANOVA followed by *q* test).

**Figure 4:**
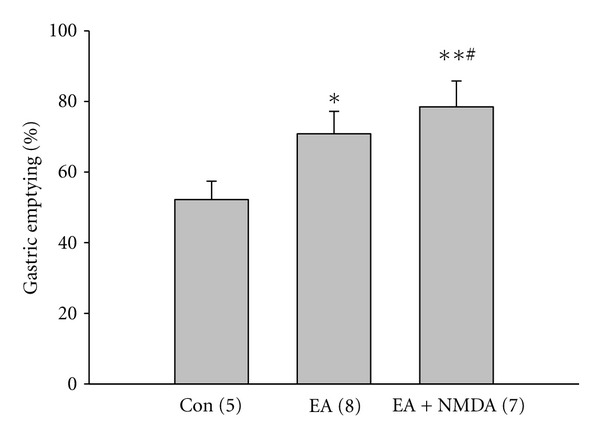
Effect of NMDA receptor (NMDAR) antagonist, kynurenic acid, on the regulation of gastric emptying by EA at BL21. EA at BL21 increased gastric emptying significantly. NMDAR agonist, NMDA, further increased gastric emptying caused by EA at BL21 (*P < 0.05, **P < 0.01, compared to the control; ^#^ *P* < 0.05, compared to EA; one-way ANOVA followed by *q* test).

To identify the role of NMDARs of DMV neurons in upregulation of gastric emptying by EA at BL21, we stereotaxically microinjected NMDAR antagonists, kynurenic acid (5 *μ*L, 0.1 mM), into DMV.  Figure 3 showed that EA increased the gastric emptying significantly (con: 58.20 ± 4.23%; EA: 72.02 ± 7.43%, *P* < 0.05). After kynurenic acid application, the increased gastric emptying by EA decreased significantly (36.59 ± 5.37%, *P* < 0.01). To further confirm whether NMDARs play a role in the upregulation of gastric emptying by EA at BL21, NMDAR agonist, NMDA (5 *μ*L, 10 *μ*M), was microinjected into DMV. As shown in Figure 4, microinjection of NMDA into DMV further increases the gastric emptying induced by EA significantly (EA ± NMDA: 88.47 ± 3.31%, *P* < 0.05). These data suggested that NMDA receptors play an important role in the upregulation of gastric emptying by EA at BL21.

### 3.4. EA at BL21 Increases NMDA Component of mEPSC but Not AMPA Component

**Figure 5:**
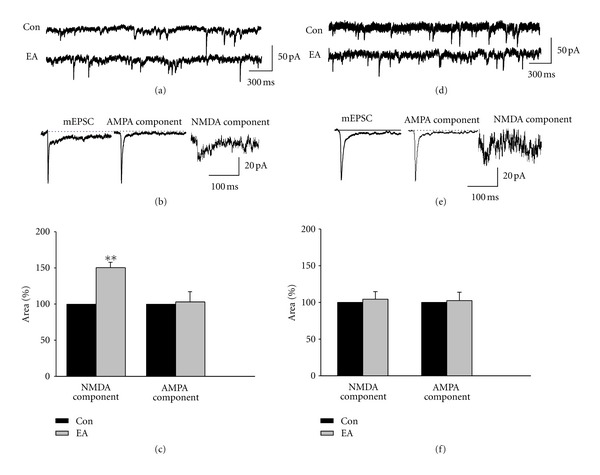
Effect of EA at BL21 on mEPSCs in labeled and unlabeled DMV neurons. (a) Representative traces of mEPSCs of labeled neurons in control and EA groups. (b) Representative AMPAR and NMDAR components of mEPSC (average over 100 events) in labeled neurons. From left to right: mEPSC containing AMPAR and NMDAR components, isolated AMPAR component, isolated NMDAR component. (c) With regard to labeled neurons in EA group, NMDAR component was increased significantly, but AMPAR component did not change significantly (**P < 0.01, unpaired *t* test, *n* = 12 neurons for each group). (d) Representative traces of mEPSCs of unlabeled neurons in control and EA groups. (e) AMPAR and NMDAR components of mEPSC (average over 100 events) in unlabeled neurons. From left to right: mEPSC containing AMPAR and NMDAR components, isolated AMPAR component, isolated NMDAR component. (f) With Regard to unlabeled neurons in both control and EA groups, neither NMDAR nor AMPAR component changed significantly (*n* = 8 neurons for each group).

Having identified that the upregulation of gastric emptying by EA at BL21 is mediated by NMDARs of DMV neurons, we went on to identify the effect of EA at BL21 on synaptic transmission in DMV neurons. To address whether EA at BL21 specifically affects the NMDAR-mediated synaptic responses in gastric-projecting DMV neurons, we first used a retrograde tracing marker to label gastric-projecting DMV neurons. Most labeled neurons were localized at the medial DMV, consistent with the previous reports [25, 27, 28]. We applied EA at BL21 for 15 min in rats with retrograde labeling and performed whole-cell recording in acute brainstem slices. Firstly we recorded mEPSCs in the acute slices and separated NMDA and AMPA component using pharmacological approach (Figures 5(a) and 5(b)). In labeled neurons, EA at BL21 did not change frequency of mEPSCs significantly (data not shown). However, as shown in Figure 5(c), EA at BL21 increased NMDA component significantly (control: 116.37 ± 15.83 pA·ms; EA: 170.72 ± 17.05 pA·ms, *P* < 0.01); in contrast, it had no significant effect on AMPA component (control: 257.05 ± 35.41 pA·ms; EA: 263.33 ± 23.70 pA·ms). Figures 5(d) and (e) showed representative traces of mEPSCs recorded in the unlabeled neurons. The frequency of mEPSC did not change significantly (data not shown). Figure 5(f) showed that EA at BL21 did not cause significant changes of either NMDA or AMPA component of mEPSCs in unlabeled neurons (NMDA component: 123.45 ± 17.37 pA*·*ms for control versus 128.35 ± 20.34 pA·ms for EA; AMPA component: 247.37 ± 27.15 pA·ms for control versus 240.42 ± 31.38 pA·ms for EA). The above results suggested that EA at BL21 enhances gastric emptying through upregulating NMDAR-mediated synaptic transmission of gastric-projecting DMV neurons.

## 4. Discussion

In the present study, we found that EA with a high intensity at BL21 increased gastric emptying in rats. NMDARs play a crucial role in this process. Enhancement of NMDAR-mediated synaptic transmission in gastric-projecting DMV neurons is required for this potent regulation of gastric emptying.

The emptying of liquids from the stomach is primarily a function of the pressure gradient between the stomach and the duodenum. Intragastric pressure is generated by gastric contractions, mainly from the proximal stomach [23, 29]. Therefore, gastric emptying of liquids seems to reflect mainly fundal activity. On the other hand, it has been generally accepted that solid gastric emptying is regulated by the coordination of the antrum, pylorus, and duodenum [29, 30]. The antral pump and pyloric opening are of paramount importance for emptying solids. Large solid particles are retained in the stomach by the pyloric closure and are retropelled and triturated in the antral mill [29, 31].

Somatovisceral reflexes responsible for regulation of visceral organs are strongly associated with the effects of acupuncture. Previous studies well documented that stimulating different skin area or points can produce different effects on gastric motility [5, 10, 11]. BL21, as one of the most common points for functional gastrointestinal disorders in the literature of traditional Chinese medicine, did not attract enough attention to its effect on these diseases. In this study, we applied EA at BL21 to observe its effect on gastric emptying and the mechanism responsible for this reaction and selected abdominal point CV12 as a control. Our results determined that EA at BL21 can mostly increase gastric emptying significantly; in contrast, EA at CV12 decreased gastric emptying significantly. Here we would like to point out that although most cases showed the upregulation of gastric emptying by BL21, in some cases EA at this point failed to accelerate or even inhibited gastric emptying, similar to the previous reports [9, 32]. What caused this phenotype will be further investigated in the future. However, regarding CV12, the results are very consistent.

 Glutamate and *γ*-aminobutyric acid (GABA) are major excitatory and inhibitory neurotransmitters within the central nervous system (CNS) [20]. DMV, as a nucleus sending efferent projections to the gastrointestinal tract, received excitatory or inhibitory information from nucleus of the solitary tract, hypothalamus, and so forth. Endogenous glutamate and GABA are the major neurotransmitters controlling the excitability of DMV motor neurons in brainstem slice preparations [28]. It is generally accepted that their inhibitory and excitatory effects on the excitability of DMV neurons are mediated directly via activation of postsynaptic GABAA receptors and both NMDA- and non-NMDA-type glutamatergic receptors, respectively [12, 33, 34]. Our current study found that NMDAR antagonist kynurenic acid abolished the acceleration of gastric emptying by EA at BL21 and agonist NMDA increased gastric emptying, indicating that EA at BL21 accelerates gastric emptying through the glutamate pathway of DMV. Our finding that EA at BL21 increased NMDAR components of mEPSC but did not change AMPA component in gastric-projecting DMV neurons suggests that NMDAR-mediated synaptic transmission of gastric-projecting DMV neurons plays a predominant role in this process.

## 5. Conclusions

In general, our works demonstrated that the upregulation of gastric emptying by EA at BL21 could be due to increasing NMDAR-mediated synaptic transmission in gastric-projecting DMV neurons.

## References

[1] Chang CS, Ko CW, Wu CY, Chen GH (2001). Effect of electrical stimulation on acupuncture points in diabetic patients with gastric dysrhythmia: a pilot study. *Digestion*.

[2] Li CH, Chung D (1976). Primary structure of human *β* lipotropin. *Nature*.

[3] Lin X, Liang J, Ren J, Mu F, Zhang M, Chen JD (1997). Electrical stimulation of acupuncture points enhances gastric myoelectrical activity in humans. *American Journal of Gastroenterology*.

[4] Ouyang H, Chen JDZ (2004). Review article: therapeutic roles of acupuncture in functional gastrointestinal disorders. *Alimentary Pharmacology and Therapeutics*.

[5] Takahashi T (2006). Acupuncture for functional gastrointestinal disorders. *Journal of Gastroenterology*.

[6] Yin J, Chen JDZ (2010). Gastrointestinal motility disorders and acupuncture. *Autonomic Neuroscience*.

[7] Li Y, Tougas G, Chiverton SG, Hunt RH (1992). The effect of acupuncture on gastrointestinal function and disorders. *American Journal of Gastroenterology*.

[8] Lux G, Hagel J, Bäcker P (1994). Acupuncture inhibits vagal gastric acid secretion stimulated by sham feeding in healthy subjects. *Gut*.

[9] Kim HY, Kwon OK, Nam TC (2000). Effect of BL-21 (Wei-Yu) acupoint stimulation on gastric motility following preanesthetic treatment in dogs. *Journal of Veterinary Science*.

[10] Noguchi E (2010). Acupuncture regulates gut motility and secretion via nerve reflexes. *Autonomic Neuroscience*.

[11] Koizumi K, Sato A, Terui N (1980). Role of somatic afferents in autonomic system control of the intestinal motility. *Brain Research*.

[12] Broussard DL, Li H, Altschuler SM (1997). Colocalization of GABA(A) and NMDA receptors within the dorsal motor nucleus of the vagus nerve (DMV) of the rat. *Brain Research*.

[13] Panico WH, Cavuto NJ, Kallimanis G (1995). Functional evidence for the presence of nitric oxide synthase in the dorsal motor nucleus of the vagus. *Gastroenterology*.

[14] Sivarao DV, Krowicki ZK, Abrahams TP, Hornby PJ (1999). Vagally-regulated gastric motor activity: evidence for kainate and NMDA receptor mediation. *European Journal of Pharmacology*.

[15] Yoon SH, Sim SS, Hahn SJ, Rhie DJ, Jo YH, Kim MS (1996). Stimulatory role of the dorsal motor nucleus of the vagus in gastrointestinal motility through myoelectromechanical coordination in cats. *Journal of the Autonomic Nervous System*.

[16] Feng HS, Lynn RB, Han J, Brooks FP (1990). Gastric effects of TRH analogue and bicuculline injected into dorsal motor vagal nucleus in cats. *American Journal of Physiology—Gastrointestinal and Liver Physiology*.

[17] Sivarao DV, Krowicki ZK, Hornby PJ (1998). Role of GABA(A) receptors in rat hindbrain nuclei controlling gastric motor function. *Neurogastroenterology and Motility*.

[18] Washabau RJ, Fudge M, Price WJ, Barone FC (1995). GABA receptors in the dorsal motor nucleus of the vagus influence feline lower esophageal sphincter and gastric function. *Brain Research Bulletin*.

[19] Browning KN, Travagli RA (2001). Mechanism of action of baclofen in rat dorsal motor nucleus of the vagus. *American Journal of Physiology—Gastrointestinal and Liver Physiology*.

[20] Iwa M, Nakade Y, Pappas TN, Takahashi T (2006). Electroacupuncture improves restraint stress-induced delay of gastric emptying via central glutaminergic pathways in conscious rats. *Neuroscience Letters*.

[21] Li YQ, Zhu B, Rong PJ, Ben H, Li YH (2007). Neural mechanism of acupuncture-modulated gastric motility. *World Journal of Gastroenterology*.

[22] Gao XY, Zhang SP, Zhu B, Zhang HQ (2008). Investigation of specificity of auricular acupuncture points in regulation of autonomic function in anesthetized rats. *Autonomic Neuroscience*.

[23] Ishiguchi T, Amano T, Matsubayashi H, Tada H, Fujita M, Takahashi T (2001). Centrally administered neuropeptide Y delays gastric emptying via Y2 receptors in rats. *American Journal of Physiology—Regulatory Integrative and Comparative Physiology*.

[24] Cheng S (1996). *Chinese Acupuncture and Moxibustion*.

[25] Browning KN, Renehan WE, Travagli RA (1999). Electrophysiological and morphological heterogeneity of rat dorsal vagal neurones which project to specific areas of the gastrointestinal tract. *Journal of Physiology*.

[26] Browning KN, Kalyuzhny AE, Travagli RA (2004). *μ*-opioid receptor trafficking on inhibitory synapses in the rat brainstem. *Journal of Neuroscience*.

[27] Krowicki ZK, Sharkey KA, Serron SC, Nathan NA, Hornby PJ (1997). Distribution of nitric oxide synthase in rat dorsal vagal complex and effects of microinjection of nitric oxide compounds upon gastric motor function. *Journal of Comparative Neurology*.

[28] Rogers RC, Hermann GE, Travagli RA (1999). Brainstem pathways responsible for oesophageal control of gastric motility and tone in the rat. *Journal of Physiology*.

[29] Minami H, McCallum RW (1984). The physiology and pathophysiology of gastric emptying in humans. *Gastroenterology*.

[30] Haba T, Sarna SK (1993). Regulation of gastroduodenal emptying of solids by gastropyloroduodenal contractions. *American Journal of Physiology—Gastrointestinal and Liver Physiology*.

[31] Brown BP, Schulze-Delrieu K, Schrier JE, Abu-Yousef MM (1993). The configuration of the human gastroduodenal junction in the separate emptying of liquids and solids. *Gastroenterology*.

[32] Kudo T, Motojima M, Kitazawa K (1991). Depression of gastric contraction by stimulation of BL-19 (Weiyu) acupoints in dogs. *American Journal of Acupuncture*.

[33] Zhang X, Fogel R (2002). Glutamate mediates an excitatory influence of the paraventricular hypothalamic nucleus on the dorsal motor nucleus of the vagus. *Journal of Neurophysiology*.

[34] Willis A, Mihalevich M, Neff RA, Mendelowitz D (1996). Three types of postsynaptic glutamatergic receptors are activated in DMNX neurons upon stimulation of NTS. *American Journal of Physiology—Regulatory Integrative and Comparative Physiology*.

